# Development and validation of a nonenhanced CT based radiomics model to detect brown adipose tissue

**DOI:** 10.7150/thno.81789

**Published:** 2023-03-05

**Authors:** Junhao Li, Rui Zuo, U. Joseph Schoepf, Joseph P. Griffith, Shiyao Wu, Changsheng Zhou, Xingzhi Chen, Weixiong Tan, Zhen Zhou, Hong Gao, Longjiang Zhang, Guifen Yang

**Affiliations:** 1 Department of Nuclear Medicine, Jinling Hospital, Medical School of Nanjing University, Nanjing 210002, China.; 2 Department of Radiology, Jinling Hospital, Medical School of Nanjing University, Nanjing 210002, China.; 3 Division of Cardiovascular Imaging, Department of Radiology and Radiological Science, Medical University of South Carolina, 25 Courtenay Dr, Charleston, SC, 29425, USA.; 4 Deepwise AI Lab, Beijing Deepwise & League of PhD Technology Co.Ltd, China.; 5 Department of Radiology, Qinhuai Medical District, Jinling Hospital, Medical School of Nanjing University, Nanjing 210002, China.

**Keywords:** Brown adipose tissues, radiomics, PET-CT, cardiovascular calcification

## Abstract

**Purpose:** It has been reported that brown adipose tissue (BAT) has a protective effect regarding cardiovascular disease. Positron emission tomography-computed tomography (PET-CT) is the reference method for detecting active BAT; however, it is not feasible to screen for BAT due to the required radionuclides and high-cost. The purpose of this study is to develop and validate a nonenhanced CT based radiomics model to detect BAT and to explore the relationship between CT radiomics derived BAT and cardiovascular calcification.

**Patients and methods:** 146 patients undergoing ^18^F-FDG PET-CT were retrospectively included from two centers for model development (n = 86) and external validation (n = 60). The data for the model development were randomly divided into a training cohort and an internal validation cohort with a 7:3 ratio, while the external validation data were divided 1:1 into a propensity score matching (PSM) cohort and a randomly sex matched cohort. Radiomics features of BAT and non-BAT depots were extracted from regions of interest (ROI) on nonenhanced CT corresponding to PET studies. Inter-class correlation coefficient (ICC) and Pearson's correlation analysis were performed to select radiomics features with high consistency. Next, least absolute shrinkage and selection operator (LASSO) with linear regression model was used to select radiomics features for model construction. Support vector machine (SVM) was used to develop the model and a radiomics score (RS) was calculated for each depot. The diagnostic performance of the radiomics model was evaluated both on a per-depot and per-patient basis by calculating the area under the receiver operating characteristic curve (AUROC). We further divided patients into BAT-RS group and non-BAT-RS group based on radiomics score and compared their cardiovascular calcification by calculating calcium volume and score.

**Results:** A total of 22 radiomics features were selected for model construction. On a per-depot basis, the AUROCs were 0.87 (95% CI: 0.83-0.9), 0.85 (95% CI: 0.79-0.90), 0.72 (95% CI: 0.67-0.77) and 0.74 (95% CI: 0.69-0.79) for detecting BAT in the training, internal validation, external validation 1 and external validation 2 cohorts, respectively. On a per-patient basis, the radiomics model had high AUROCs of 0.91 (95% CI: 0.84-0.98), 0.77 (95% CI: 0.61-0.92) and 0.85 (95% CI: 0.72-0.98) in the training, external validation 1 and external validation 2 cohorts, respectively. When grouping based on the radiomics model, the BAT-RS group had lower odds of coronary artery calcium (CAC) and thoracic aorta calcium (TAC) compared with the non-BAT-RS group (CAC: 2.8% *vs.* 20.3%, p = 0.001; TAC: 19.4% *vs.* 39.2%, p = 0.009). The BAT-RS group had less CAC volume (4.1 ± 4.0 mm^3^ *vs.* 147.4 ± 274.3 mm^3^; p = 0.001), CAC score (2.8 ± 3.0 *vs.* 169.1 ± 311.5; p = 0.001), TAC volume (301.4 ± 450.2 mm^3^ *vs.* 635.3 ± 1100.7 mm^3^; p = 0.007) and TAC score (496.2 ± 132.6 *vs.* 749.2 ± 1297.3; p = 0.007) than the non-BAT-RS group.

**Conclusion:** We developed and validated a nonenhanced CT based reliable radiomics model for detecting BAT with PET-CT findings as reference standard. Radiomics signatures from nonenhanced CT can reliably detect BAT and have promising potential to be used in routine clinical settings. Importantly, our study showed that patients with BAT had less cardiovascular calcification.

## Introduction

With an increasing proportion of the population aging, cardiovascular disease remains the leading cause of disease burden around the world 1. Adipose tissue, like pericoronary adipose tissue and epicardial adipose tissue, were regarded as biomarkers of inflammation and may aggravate atherosclerosis in a paracrine way 2, 3. However, brown adipose tissue (BAT), which has a non-shivering thermogenic function, has recently been shown to have a protective effect on the cardiovascular system 4, 5. Subjects with BAT have a low prevalence of coronary artery disease, lower levels of conventional cardiovascular risk factors at baseline, lower carotid intima-media thickness and higher carotid elasticity at 5-year follow-up, and its benefit was more significant in the obese population 6-8. Considering this, screening for BAT in the general population may improve the prevention and control of cardiovascular disease.

Currently, ^18^F-fluorodeoxyglucose (FDG) PET-CT is the conventional method and reference standard to detect BAT 9. However, ^18^F-FGD PET-CT is unlikely to be used to screen for BAT in the general population, as the need for an onsite cyclotron facility for generating radionuclides, the short physical half-life of nuclides and the high cost of PET imaging. Thus, to achieve this goal, we need to develop a widely available and low-cost technique for BAT detection. Nonenhanced CT has been widely used in screening for a broad range of conditions and is an ideal modality to screen for BAT in a general population 10, 11. Additionally, a single nonenhanced chest CT scan can anatomically cover most of BAT depots, as they are mainly distributed in cervical, supraclavicular, axillary and mediastinal regions 4, 5, 12. However, it is rather difficult to distinguish BAT from white adipose tissue in conventional nonenhanced CT images. BAT has different histological features from white adipose tissue. Specifically, BAT contains small lipid droplets and high mitochondrial content in a single cell, with high vascularization and rich sympathetic innervation on gross pathology 13, 14. Based on the histological differences in composition and structure, CT based radiomics may be a promising way for BAT detection. Radiomics could be used to capture subtle differences in intensity and texture from the images as well as analyze tissue features at the raw-data level, which may reveal more characteristics than those visible on the original images 15. Therefore, a combination of nonenhanced CT and radiomics would be an optimal way to screen for BAT.

Our hypothesis was that nonenhanced CT based radiomics can detect BAT and that BAT correlates with cardiovascular health. Thus, the main aim of this study was to develop and validate a radiomics model to detect BAT on nonenhanced CT and to explore the association of BAT with cardiovascular calcification.

## Materials and Methods

### Patients

Patients who underwent a whole-body PET-CT scan and incidentally found BAT were retrospectively included in this study. We collected patients' clinical information including age, sex, weight, and outdoor temperature on the day of examination. Based on the brown adipose reporting criteria in imaging studies (BARCIST 1.0), BAT was confirmed when meeting both following criteria: 1) A CT Hounsfield unit (HU) range from -190 to -10 HU to identify adipose tissue on CT image; 2) A standard uptake value at the basal metabolic rate (SUV_bm_) ≥ 1.5 to identify a high metabolic state of adipose tissue 9. Exclusion criteria included: 1) Enlarged lymph nodes or lesions in cervical, supraclavicular, axillary or mediastinal depots; 2) Active vasculitis or systemic inflammation; 3) History of medications that may interfere with BAT, such as beta blockers; 4) Poor quality PET-CT images or missing clinical/image data. The control subjects were defined as patients who underwent a whole-body PET-CT scan and found no BAT, with the same exclusion criteria of the BAT patients.

For the model development cohort (training and internal validation cohort), we retrospectively collected patients with BAT from Jinling Hospital, Medical School of Nanjing University (Nanjing, China). Additionally, patients with BAT were retrospectively included from Qinhuai Medical District, Jinling Hospital, Medical School of Nanjing University (Nanjing, China) for external validation (external validation cohort 1 and external validation cohort 2). To minimize the impact of other factors, 1:1 propensity score matching (PSM) based on age, sex, weight and outdoor temperature of the scan was applied to match controls in the model development cohort and the external validation cohort 1. To further assess the generalizability of the model, an equal number of controls matched by sex were randomly selected in the external validation cohort 2.

**Figure 1:**
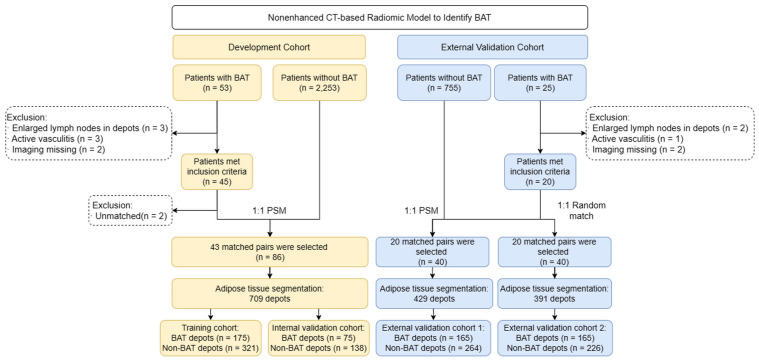
** Study flowchart.** BAT = brown adipose tissue; PSM = propensity score matching

Finally, 50 patients with BAT in Jinling Hospital were screened but 7 patients were excluded. The remaining 43 patients with BAT collected from Jinling Hospital met the BAT criteria and matched with 43 control patients by 1:1 PSM in the development cohort. In external validation cohorts, 23 patients with BAT in Qinhuai Medical District were screened but 3 patients were excluded. The remaining 20 patients with BAT collected from Qinhuai Medical District met the BAT criteria. They were matched with 20 controls by 1:1 PSM to set up external validation cohort 1. Besides, they were also matched with another 20 controls by 1:1 randomly matching by sex to set up external validation cohort 2. The study flowchart is shown in **Figure 1**. The ethics committee of Jinling Hospital approved the study protocol, and the need for written informed patient consent was waived.

### Image acquisition

PET-CT examinations of the development and external validation cohorts were performed, respectively, on two PET-CT scanners, and thin-section chest CT scans were performed subsequently on the same PET-CT scanner. In the development cohort, PET-CT scans were obtained using Biogragh 16 scanner (Siemens, Erlangen, Germany) (n = 86), while in external validation cohorts, PET-CT scans were obtained using Biogragh 16 HR scanner (Siemens, Erlangen, Germany) (n = 60). The patients fasted for at least 6 hours before the examination. The blood glucose levels of patients were controlled to < 6.7 mmol/L before intravenous ^18^F-FDG injection. The intravenous injection dose of ^18^F-FDG was 3.7-6.6 MBq/kg. After the injection, patients rested quietly for one hour before scanning. The scan ranged from the skull base to the upper femur. Detailed scan parameters were as follows: 1) Biogragh 16: CT scan parameters: slice thickness = 5 mm, pitch = 0.75, tube voltage = 120 kVp, effective tube current = 290 mAs, tube rotation speed = 0.8 s/rot, matrix = 512×512. PET scan parameters: 3 min per bed position. 2) Biogragh 16 HR: CT scan parameters: slice thickness = 5 mm, pitch = 1.25, tube voltage = 120 kVp, effective tube current = 320 mAs, tube rotation speed = 0.8 s/rot, matrix = 512×512. PET scan parameters: 3 min per bed position. CT images were reconstructed with B31f convolution kernel and iterative algorithm. PET images were attenuation corrected based on CT, and an iterative algorithm was used for image reconstruction. Additionally, thin-section chest CT was acquired with the following parameters: slice thickness = 2 mm, pitch = 0.75, tube voltage = 120 kVp, effective tube current = 200 mAs, gantry rotation speed = 0.8 s/rot; matrix=512×512. Convolution kernel B31f and an iterative algorithm were used for image reconstructions. Thin-section chest CT images were reconstructed with 3 mm slice thickness for calcium score calculation.

### Vascular calcification assessment

Thin-section chest CT images were transferred to an offline workstation (Syngo.via, Siemens Healthineers) for vascular calcium assessment. CAC score was calculated on noncontrast noncardiac CT according to the guidelines of the Society of Cardiovascular Computed Tomography and the Society of Thoracic Radiology 16. A cardiovascular radiologist (R.Z. with 2 years of experience in interpreting cardiovascular CT) evaluated the calcification of coronary arteries and/or thoracic aorta in nonenhanced CT images using Syngo.via Calcium Scoring (VB20A, Siemens). At least three adjacent pixels with densities >130 HU were considered calcium. The calcium volume and score of detected calcifications of the coronary arteries and thoracic aorta were calculated based on the Agatston score 17.

### Radiomics analysis

#### Adipose tissue segmentation

PET-CT images were transferred to the Deepwise Multimodal Research Platform (version 2.0, Beijing Deepwise & League of PHD Technology Co., Ltd, Beijing, China, https://keyan.deepwise.com), an online platform for medical data processing. ROIs were drawn in both BAT patients and control patients. All adipose tissue depots possibly containing BAT were manually delineated on the Deepwise Multimodal Research Platform, including bilateral symmetrical cervical, supraclavicular, axillary depots and asymmetrical mediastinal depots 4, 5, 12. The target adipose tissues were segmented slice by slice from the skull base to the bifurcation of the trachea. Because the heart is a hypermetabolic organ, pericardial fat often presents with a high SUV_bm_ that interferes with the diagnosis of BAT. Thus, the mediastinal depot below the aortic arch level was disregarded. A detailed segmentation process is shown in **Figure S1**. Each patient had 6-13 depots. Additionally, the reader (J.H.L., with 3 years' experience in CT interpretation) was blinded to PET-CT findings during the process of segmentation to reduce measurement bias. When all of the adipose tissue depot segmentations were finalized, the depots were labelled as BAT or non-BAT according to corresponding PET-CT findings. In the development cohort, a total of 250 BAT depots and 459 non-BAT depots were segmented from 86 patients (BAT, n = 43 patients, non-BAT, n = 43 patients). Both BAT and non-BAT depots were randomly divided into a training set and a validation set with a 7:3 ratio, respectively. In the external validation cohort 1, 165 BAT depots and 264 non-BAT depots were segmented from 40 patients (BAT, n = 20 patients, non-BAT, n = 20 patients). In the external validation cohort 2, 165 BAT depots and 226 non-BAT depots were segmented from 40 patients (BAT, n = 20 patients, non-BAT, n = 20 patients).

#### Feature extraction, selection and model construction

**Figure 2:**
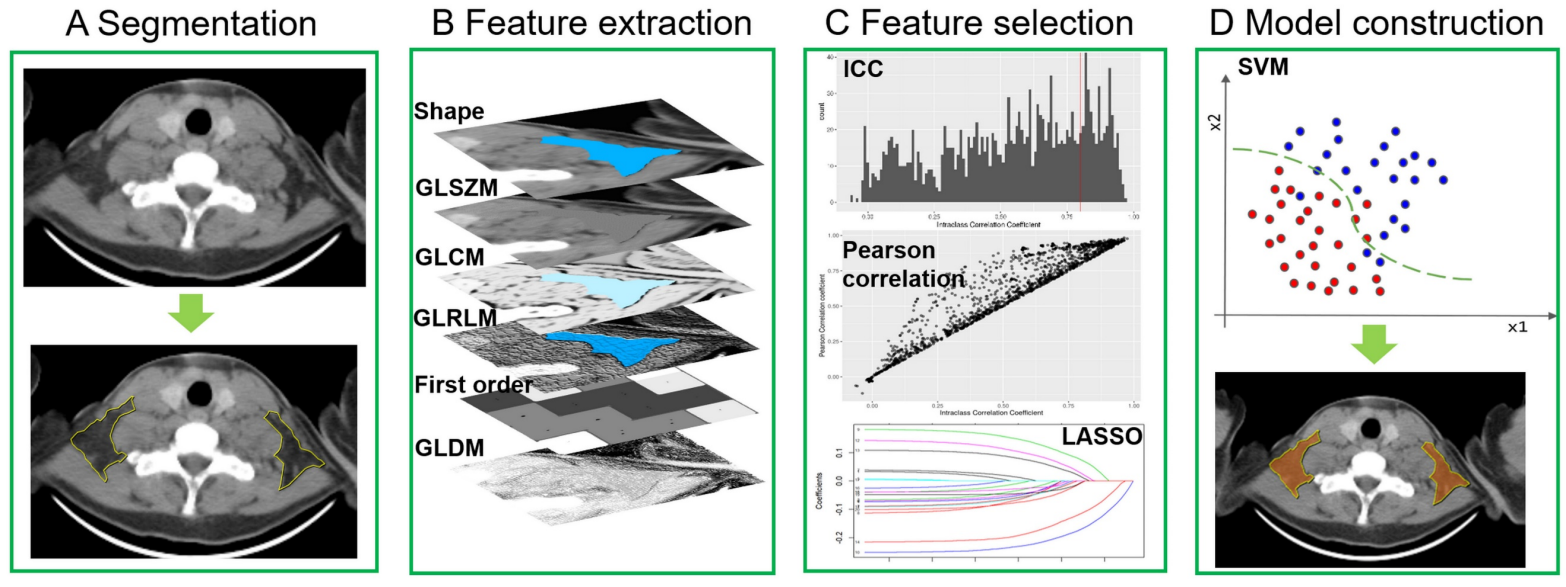
** Radiomics model development.** GLSZM = gray-level size zone matrix; GLCM = gray-level co-occurrence matrix; GLRLM = gray-level run-length matrix; GLDM = gray-level dependence matrix; ICC = inter-class correlation coefficient; LASSO = least absolute shrinkage and selection operator; SVM = support vector machine.

The process of radiomics features extraction, selection and model construction were all performed on the Deepwise Multimodal Research Platform (version 2.0, Beijing Deepwise & League of PHD Technology Co., Ltd, Beijing, China, https://keyan.deepwise.com). All images were resampling to [1,1,0] after B-spline interpolation sampling technology conducted. The window-center and window-width were set at [40, 400] HU. Before feature extraction, 10 types of image filters, a mathematical processing, were used to perform image processing. Then radiomics features of BAT and non-BAT were extracted from the segmented ROI in axial CT images with a 5 mm slice thickness. Based on original images and different filtered image filters (10 types of images filtering methods shown in **Table S1)** and feature groups (7 groups), we extracted 1,743 radiomics features from the segmented depots including 342 first-order features based on the voxel intensity, 14 shape features, and 1387 textures features (418 gray-level co-occurrence matrix (GLCM), 304 gray-level size zone matrix (GLSZM), 304 gray-level run-length matrix (GLRLM), 266 gray-level dependence matrix (GLDM) and 95 neighboring gray tone difference matrix (NGTDM)). Inter-class correlation coefficient (ICC) was then performed to evaluate the interobserver reproducibility of all extracted features based on segmentation, and those with an ICC <0.80 were excluded. After the exclusion, only 108 radiomics features remained. Additionally, Pearson's correlation analysis was also applied to filter out redundant radiomic features, which removed 86 features. For example, when 2 features were highly correlated (Pearson's correlation coefficient >0.90), the one with the lower correlation coefficient with BAT was removed. The remaining features were further selected by various feature selections with a basic model of support vector machine (SVM) algorithm, and least absolute shrinkage and selection operator (LASSO) with linear regression model had the highest area under the receiver operating characteristic curve (AUROC) (**Table S2**). Finally, 22 radiomics features were selected for model construction. They were grouped as follows: 1) 9 first order features, 2) 3 gray-level co-occurrence matrix features, 3) 3 gray-level size zone matrix features, 4) 3 gray-level run-length matrix features, 5) 1 gray-level dependence matrix features and 6) 3 shape features. The details of selected features were listed in **Table S3**. We developed the model using various machine learning and support vector machine (SVM) algorithms had the highest AUROC (**Table S2**). Therefore, we chose the SVM algorithm to develop the model and computed a BAT radiomics score (BAT-RS) for each depot. The process of the radiomics model development is shown in **Figure 2**.

#### BAT diagnosis based on the radiomics model

On a per depot basis, each depot was defined as a BAT or non-BAT depot based on the cut-off threshold of the radiomics score. The optimal cut-off value was determined using Youden's index in the development cohort.

On a per patient basis, we set two different diagnosis criteria to define BAT positive patient according to the results of depot detected by the radiomics model. In criterion 1, patients harboring at least one BAT depot detected by the radiomics model were considered BAT positive. In criterion 2, to reduce the false positive rate, the visually symmetrical physiologic distribution pattern of the depots defined by the radiomics model was considered BAT positive. Numerous studies have demonstrated that BAT is symmetrically distributed in cervical, supraclavicular and axillary regions 1, 5, 11. Thus, BAT positive patients were considered to have at least one pair of symmetrically distributed BAT-RS positive depots in cervical, supraclavicular or axillary depots or one mediastinal depot. Examples are shown in **Figure S2**.

### Statistical analysis

Statistical analysis was performed with SPSS Statistics version 26.0 (IBM SPSS Inc.) and MedCalc software version 19.0.7.0 (MedCalc Software). For continuous variables, normal distribution was firstly tested using the Kolmogorov-Smirnov test. According to the data distribution, continuous variables were presented as a mean ± SD or median (25^th^, 75^th^). The Student t-test or Mann-Whitney test was used for the comparison between the BAT group and control group as appropriate. For categorical variables, data were presented as frequencies (percentages). The comparison between the two groups was performed with a Chi-squared test or a Fisher's exact test, as appropriate. The correlations between parameters were analyzed using Pearson correlation analysis. 1:1 propensity score matching using logistic regression with a nearest-neighbor caliper width of 0.1 was used to match the control group in model development cohort and external validation cohort 1. The Youden index was used to determine the optimal cut-off values for determination of a BAT depot in the development set. The diagnostic performance of the radiomics model was evaluated both on a per depot level and per patient basis by sensitivity, specificity, positive predictive value (PPV), negative predictive value (NPV), accuracy and AUROC with 95% confidence intervals (95% CI). Besides, we calculated AUROC-cutoff for binary prediction (based on the optimal cut-off values of radiomic score). A value of p < 0.05 (2 tailed) was considered statistically significant.

## Results

### Clinical characteristics

The clinical characteristics of the model development cohort and external-validation cohorts are presented in **Table 1**. In the model development cohort and external validation cohort 1, no significant differences were noted between the BAT group and the control group (all p > 0.05). In the external-validation cohort 2, the patients in the BAT group were younger than the control group (40 [28, 47] years *vs.* 53 [48, 56] years, p < 0.001). Additionally, the outdoor temperature in the BAT group was higher than in the control group (highest temperature: 13 [9, 19] ℃ *vs.* 5 [3, 6] ℃, p < 0.001; lowest temperature: 4 [0, 13] ℃ *vs.* -2 [-3, 2] ℃, p < 0.001). Sex and body weight showed no statistical difference between the BAT group and the control group. The incidence of BAT had a negative correlation with the outdoor highest and lowest temperature (r=-0.699, p = 0.025 and r = -0.727, p = 0.017, respectively) (**Figure S3**). We found that the mean volume of BAT depots in axilla was smaller than that of non-BAT depots in all cohorts (Development cohort: 62.4 ± 39.4 cm^3^ *vs.* 85.8 ± 38.8 cm^3^, p = 0.012; External validation cohort 1: 47.7 ± 13.2 cm^3^ *vs.* 69.7 ± 34.0 cm^3^, p = 0.022; External validation cohort 2: 47.7 ± 13.2 cm^3^ *vs.* 84.2 ± 22.7 cm^3^, p < 0.001). Besides, the mean volume of BAT depots in neck, axilla and mediastinum was smaller than that of non-BAT depots in external validation cohort 2 (Cervical: 8.9 ± 7.5 cm^3^ *vs.* 16.6 ± 6.9 cm^3^, p < 0.001; Axillary: 47.7 ± 13.2 cm^3^ *vs.* 84.2 ± 22.7 cm^3^, p < 0.001; Mediastinal: 6.3 ± 6.2 cm^3^ *vs.* 9.3 ± 6.3 cm^3^, p = 0.041).

### Diagnostic performance of the radiomics model

The optimal cut-off value of BAT-RS was 0.30. Thus, depots with a radiomics score > 0.30 were regarded as BAT-RS positive. The diagnostic performance of the radiomics model in the training, internal validation, and external validation cohorts is shown in **Table 2**. On a per depot level, the depots segmented from each patient in development cohort were randomly divided into training set and internal validation set. Thus, there are four datasets on a per depot level, i.e., training set, internal validation set, external validation cohorts 1 and 2. We did not divide the patients into training set and internal validation set in development cohort due to small sample size. Thus, there are three datasets on a per patient level, training set, external validation cohorts 1 and 2. On a per depot basis in the training and internal validation cohorts, the radiomics model detected BAT well based on the radiomic score, with AUROCs of 0.87 (95% CI: 0.83, 0.90) and 0.85 (95% CI: 0.79, 0.90), respectively. In the external validation cohorts 1 and 2, the model yielded AUROCs of 0.72 (95% CI: 0.67, 0.77) and 0.74 (95% CI: 0.69, 0.79), respectively. Besides, we also evaluated the diagnostic performance of the model based on the optimal cut-off value (RS=0.30), and it performed stably with an AUROC-cutoff of 0.80 (95% CI: 0.76, 0.84), 0.76 (95% CI: 0.69, 0.81), 0.68 (95% CI: 0.64, 0.73) and 0.70 (95% CI: 0.65, 0.75), respectively. On a per patient basis, we set two different criteria to define BAT positive patients detected by the radiomics model. With criterion 1, the model presented the highest sensitivity but low specificity and AUROC-cutoff in the training cohort and the external validation cohorts 1 and 2 (sensitivity: 97.7%, 95% and 85%; specificity: 60.5%, 30% and 40%; accuracy: 79.1%, 62.5% and 62.5%; AUROC-cutoff: 0.79, 0.63 and 0.63).

To improve specificity, we incorporated the physiological distribution of BAT into the diagnostic model with criterion 2. The addition of criterion 2 significantly improved the specificity, accuracy and AUROC-cutoff, and the sensitivity was only slightly reduced (sensitivity: 97.7%, 80% and 80%; specificity: 83.7%, 70% and 90%; accuracy: 90.7%, 75% and 85%; AUROC-cutoff: 0.91, 0.77 and 0.85).

### Comparison of cardiovascular calcification between the control and the BAT/BAT-RS and non-BAT-RS groups

The BAT group, as determined by PET-CT, had a significantly lower incidence of CAC (1.6% *vs.* 19.3%, p =0.001) and TAC (20.6% *vs.* 36.1%, p = 0.042) compared to the control group. Additionally, both the calcium volume and score of the BAT group were lower than those of the control group (CAC volume: 6.9 ± 0 mm^3^ *vs.* 138.2 ± 267.5 mm^3^, p = 0.001; TAC volume: 269.2 ± 446.9 mm^3^ *vs.* 638.2 ± 1082.4 mm^3^, p = 0.029; CAC score: 4.9 ± 0 *vs.* 158.6 ± 303.8; p = 0.001; TAC score: 306.0 ± 482.4 *vs.* 753.5 ± 1276.3, p = 0.028) (**Table 3**). The values for cardiovascular calcification of each cohort are presented in **Table 1.**

**Figure 3:**
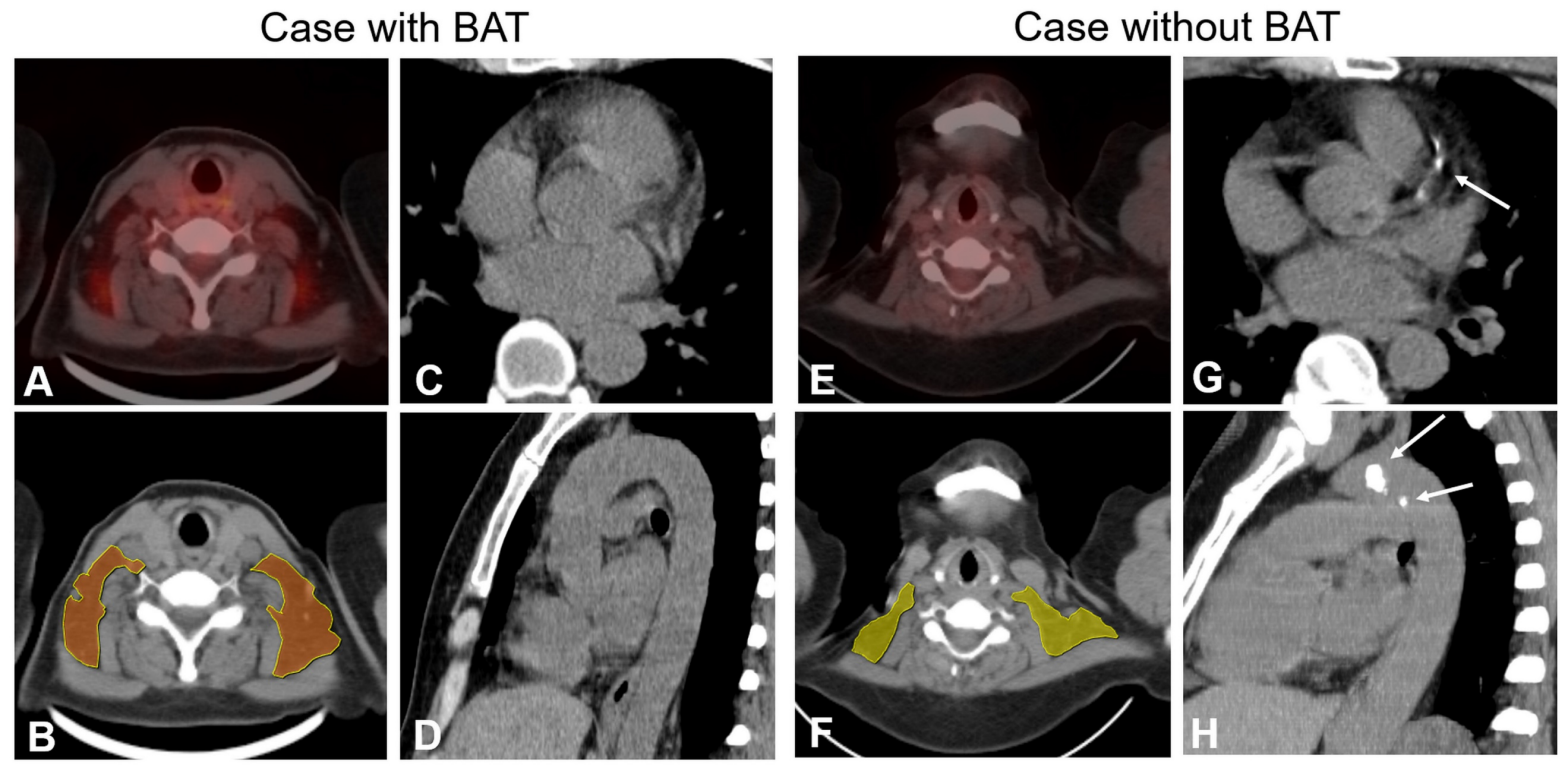
** Examples of patients with and without BAT and cardiovascular calcification.** PET-CT finding (**panel A**) and radiomics model (**panel B**) show BAT in bilateral supraclavicular depots in a 65-year-old female. Axial and sagittal CT images (**Panels C** and **D**) show no calcification of coronary arteries and thoracic aorta. PET-CT finding and radiomics model (**Panels D** and **F**) show no BAT depots in a 51-year-old female. Axial and sagittal CT images (**Panels G** and **H)** show calcified plaques both in the coronary arteries and thoracic aorta (white arrow).

The BAT-RS group, as determined by the CT radiomics model, had lower odds of CAC and TAC compared with the non-BAT-RS group (CAC: 2.8% *vs.* 20.3%, p = 0.001; TAC: 19.4% *vs.* 39.2%, p = 0.009). The BAT-RS group had less CAC volume (4.1 ± 4.0 mm^3^ *vs.* 147.4 ± 274.3 mm^3^; p = 0.001) and a lower CAC score (2.8 ± 3.0 *vs.* 169.1 ± 311.5; p = 0.001). The difference in thoracic aorta calcification was more significant. There was lower TAC volume (301.4 ± 450.2 mm^3^ *vs.* 635.3 ± 1100.7 mm^3^; p = 0.007) and a lower TAC score (496.2 ± 132.6 *vs.* 749.2 ± 1297.3; p = 0.007) in the BAT-RS group compared to the non-BAT-RS group (**Table 3**). The values for cardiovascular calcification of each cohort are presented in **Table S4.** Examples of patients with and without CT radiomics based BAT and their cardiovascular calcifications are presented in **Figure 3.**

## Discussion

We developed and validated a BAT-RS model based on nonenhanced CT images, which demonstrated good diagnostic performance in both development and external validation cohorts. To the best of our knowledge, this is the first study to develop and validate a radiomics model for diagnosing BAT based on nonenhanced CT images.

Previous studies have explored BAT detection methods based on magnetic resonance imaging (MRI) or infrared thermography 18-21. However, these studies demonstrated a low AUROC (0.25-0.68) and failed to determine an optimal cut-off value to diagnose BAT. Detecting BAT based on nonenhanced CT by radiomics would be a potentially suitable way to screen for BAT in the general population. A previous pilot study by Nazeri *et al.* presented the feasibility of detecting BAT using a CT based radiomics model 22. They extracted BAT radiomics features from both PET and low-dose CT images of 18 healthy adults and found 6 CT radiomics features with high repeatability 22. However, to the best of our knowledge, no radiomics model has been developed and validated to test the performance of CT based radiomics in detecting BAT. In our study, we developed a BAT radiomics model based on nonenhanced CT images, which had reliable performance in both the development and external-validation cohorts. We found a total of 211 CT radiomics features with high repeatability, which was substantially more than in the previous study by Nazeri *et al.* We hypothesize that, because our study extracted and analyzed radiomics features based on a per depot level, there was a relatively large sample size of radiomics features, which accounts for the greater number of reliable radiomics features seen in our study. We are the first to evaluate the performance of a radiomics model, both on a per depot and per patient basis. Notably, our study incorporated the physiologic distribution pattern of BAT into the diagnostic model on a per patient basis, which significantly improved the specificity and accuracy in each cohort by reducing false positive results. The results demonstrate the feasibility of a BAT diagnostic model that utilizes a combination of radiomics features and the physiological distribution of BAT.

In addition to the development and validation of a radiomics model, we also explored the relationship between BAT and calcium score based on both PET-CT findings and the radiomics model. When grouped based on the PET-CT findings, the BAT group showed a lower incidence of CAC and TAC, while the calcium volume and score were all lower than in the control group. Several basic and clinical studies have demonstrated that BAT has a protective effect on the cardiovascular system 6, 23. Specifically, Nam *et al.* found that BAT was associated with a low CAC 7. Our results showed that the differences of calcium volume and score were more significant between the BAT-RS group and the non-BAT-RS group. We hypothesize that our model identified some non-active BAT, accounting for the larger difference. Due to the lack of cold stimulation, these BAT depots without activation would be falsely classified as non-BAT depots when only analyzed by PET-CT. Our radiomics model detected these non-active depots as BAT depots, and thus the difference in the cardiovascular calcification was more significant. Based on these findings, our nonenhanced CT based radiomics model made BAT screening possible without the additional need for cold stimulation. Therefore, our model offers a promising way to screen for BAT. However, the method needs to be further verified in the future.

Our study has several limitations. First, some important clinical parameters were not collected such as height and diabetes due to the retrospective nature of the study, and their impact on BAT could not be controlled. Second, we manually segmented all adipose tissue depots, which is time and labor consuming and can introduce segmentation measurement error. For example, a segmented BAT depot consisted of not only brown adipose tissue but also a part of white adipose tissue, which would affect the diagnostic performance of the radiomics model. A precise algorithm for BAT segmentation and volume calculation needs to be developed. Third, as a retrospective and observational study, the patients did not uniformly undergo cold stimulation, meaning that some BAT depots without activation could be classified as non-BAT depots. A modified segmentation and suitable cold stimulation should be applied in a future study to extract the radiomics features of BAT more precisely. Fourth, we developed and validated a radiomics model with a relatively small size sample, especially for the external cohorts. Finally, we used binary patient-based BAT analysis in this study. Although it performed well, quantification of BAT with a cut-off value will be a much stronger imaging marker for guiding the patients' prognosis. Taken together, further studies are needed to resolve these issues.

## Conclusions

In conclusion, we developed and validated a BAT detection model based on CT radiomics features that performed favorably. Our study suggests that a nonenhanced CT-based radiomics model is a feasible and convenient method for BAT screening. Our study also highlights that patient with BAT had better cardiovascular health, as indicated by lower CAC and TAC volumes and scores.

## Supplementary Material

Supplementary figures and tables.

## Figures and Tables

**Table 1:** Clinical characteristics and vascular calcification of included patients

Variables	All	Model Development Cohort				External-Validation Cohort 1				External-Validation Cohort 2	
		BAT	1:1 PSM Control	P value		BAT	1:1 PSM Control	p value		Random Control	p value
**Patients,** n	146	43	43	-		20	20	-		20	-
**Age,** years	43 ± 12	42 ± 11	44 ± 14	0.365		37 ± 12	41 ± 11	0.304		53 (48, 56)	<0.001*
**Sex**				0.645				0.527			>0.999
Male, n (%)	57 (39.0)	13 (30.2)	15 (34.9)			9 (45.0)	11 (55.0)			9 (45.0)	
Female, n (%)	89 (61.0)	30 (69.8)	28 (65.1)			11 (55.0)	9 (45.0)			11 (55.0)	
Weight, kg	59.8 (52.0, 65.0)	60.0 (56.0, 65.0)	60.0 (55.4, 65.0)	0.628		57.0 (50.5, 67.0)	65.0 (50.5, 73.0)	0.525		64.5±10.9	0.136
**Temperature, °C**											
Highest	12.0 (5.0, 17.0)	14.0 (9.0, 19.0)	10.0 (4.0, 17.0)	0.084		13.8 ± 7.2	12.3 ± 6.6	0.482		5.0 (3.0, 6.0)	0.021*
Lowest	4.0 (0, 10.0)	5.0 (0, 10.0)	5.0 (2.0, 14.0)	0.516		6.0 ± 8.1	5.4 ± 5.3	0.782		-2.0 (-3.0, 2.0)	0.004*
**Depot volume^a^, cm^3^**											
Cervical	14.4 ± 9.7	18.2 ± 13.3	16.9 ± 10.2	0.612		8.9 ± 7.5	11.7 ± 7.1	0.067		16.6 ± 6.9	<0.001*
Supraclavicular	41.1 ± 26.5	33.1 ± 16.2	36.2 ± 16.3	0.371		28.0 ± 17.3	33.9 ± 24.5	0.119		31.5 ± 18.5	0.414
Axillary	76.9 ± 35.6	62.4 ± 39.4	85.8 ± 38.8	0.012*		47.7 ± 13.2	69.7 ± 34.0	0.022*		84.2 ± 22.7	<0.001*
Mediastinal	8.8 ± 6.7	10.2 ± 7.8	9.1 ± 5.6	0.391		6.3 ± 6.2	7.8 ± 6.1	0.308		9.3 ± 6.3	0.041*
**Vascular calcification**											
CAC > 0, n (%)	17 (11.6)	1 (2.3)	8 (18.6)	0.035*		0 (0)	2 (10.0)	0.487		6 (30.0)	0.020*
TAC > 0, n (%)	43 (29.5)	9 (20.9)	14 (32.6)	0.223		4 (20.0)	5 (25.0)	1.000		11 (55.0)	0.022*
CAC volume^b^, mm^3^	130.5 ± 261.0	6.9 ± 0	259.8 ± 345.4	0.013*		0	24.4 ± 25.5	0.152		14.1 ± 13.9	0.009*
TAC volume^b^, mm^3^	526.6 ± 946.3	340.9 ± 528.0	918.1 ± 1098.9	0.145		108.0 ± 74.2	916.8 ± 1863.2	0.779		155.2 ± 184.5	0.032*
CAC score^b^	149.6 ± 296.6	4.9 ± 0	297.5 ± 391.6	0.013*		0	28.3 ± 29.7	0.152		16.8 ± 19.0	0.009*
TAC score^b^	618.2 ± 1111.1	384.1 ± 568.9	1107.2 ± 1333.7	0.139		130.2 ± 91.3	1032.4 ± 2113.3	0.799		176.5 ± 212.5	0.032*

Values presented as mean ± SD, n (%) or median (interquartile range). BAT = Brown adipose tissue; PSM = Propensity score matching; CAC = Coronary artery calcium; TAC = Thoracic aorta calcium ^a^Only BAT depots were used for depot volume calculation, ^b^Only calcium positive was presented, but all patients were accepted for P value calculation. * p value < 0.05.

**Table 2:** Performance of the radiomics model on a per depot level and a per patient level.

Data	Sensitivity, %	Specificity, %	PPV, %	NPV, %	Accuracy, %	AUROC^a^	AUROC-cutoff^b^
**On a per depot level**							
**Training**	86.9 (80.9-91.5)	73.5 (68.3-78.3)	64.1 (57.6-70.2)	91.1 (86.8-94.2)	78.2 (74.3-81.7)	0.87 (0.83-0.90)	0.80 (0.76-0.84)
**Internal**	89.3 (80.1-95.3)	61.6 (52.9-69.7)	55.8 (46.5-64.8)	91.4 (83.3-95.9)	71.4 (64.7-77.2)	0.85 (0.79-0.90)	0.76 (0.69-0.81)
**External 1**	78.2 (71.0-84.1)	57.6 (51.4-63.6)	53.5 (47.0-59.9)	80.9 (74.3-86.1)	66.1 (61.4-70.5)	0.72 (0.67-0.77)	0.68 (0.64-0.73)
**External 2**	78.8 (71.8-84.8)	58.7 (52.5-64.6)	60.0 (53.1-66.5)	79.5 (72.7-85.1)	68.8 (63.9-73.3)	0.74 (0.69-0.79)	0.70 (0.65-0.75)
**On a per patient level**							
**Diagnosis criterion 1**							
**Training**	97.7 (86.2-99.9)	60.5 (44.4-74.6)	71.2 (57.7-81.9)	96.3 (79.1-99.8)	79.1 (68.7-86.8)		0.79 (0.69-0.89)
**External 1**	95.0 (73.1-99.7)	30.0 (12.8-54.3)	57.6 (39.4-74.0)	85.7 (42.0-99.2)	62.5 (45.8-76.8)		0.63 (0.46-0.81)
**External 2**	85.0 (61.1-96.0)	40.0 (20.0-63.6)	58.6 (39.1-75.9)	72.7 (39.3-92.7)	62.5 (45.8-76.8)		0.63 (0.45-0.80)
**Diagnosis criterion 2**							
**Training**	97.7 (86.2-99.9)	83.7 (68.7-92.7)	85.7 (72.1-93.5)	97.3 (84.2-99.9)	90.7 (82.0-95.6)		0.91 (0.84-0.98)
**External 1**	80.0 (55.7-93.4)	70.0 (45.7-87.2)	72.7 (49.6-88.4)	77.8 (51.9-92.6)	75.0 (58.5-86.8)		0.77 (0.61-0.92)
**External 2**	80.0 (55.7-93.4)	90.0 (66.9-98.2)	88.9 (63.9-98.1)	81.2 (59.0-94.0)	85.0 (69.5-93.8)		0.85 (0.72-0.98)

^a^AUROC for the radiomic score (regression model prediction); ^b^AUROC for the optimal cut-off value (Radiomic score=0.30) (binary prediction); PPV = positive predictive value; NPV = negative predictive value; AUROC = area under the receiver operating characteristic curve

**Table 3:** Comparisons of cardiovascular calcification between the control and the BAT/BAT-RS and non-BAT-RS groups.

Variables	All	BAT	Control	p value	BAT-RS	Non-BAT-RS	p value
**Patients, n**	146	63^a^	83^b^	-	72	74	-
**CAC > 0, n (%)**	17 (11.6)	1 (1.6)	16 (19.3)	0.001*	2 (2.8)	15 (20.3)	0.001*
**TAC > 0, n (%)**	43 (29.5)	13 (20.6)	30 (36.1)	0.042*	14 (19.4)	29 (39.2)	0.009*
**CAC volume^#^, mm^3^**	130.5 ± 261.0	6.9 ± 0	138.2 ± 267.5	0.001*	4.1 ± 4.0	147.4 ± 274.3	0.001*
**TAC volume^#^, mm^3^**	526.6 ± 946.3	269.2 ± 446.9	638.2 ± 1082.4	0.029*	301.4 ± 450.2	635.3 ± 1100.7	0.007*
**CAC score^#^**	149.6 ± 296.6	4.9 ± 0	158.6 ± 303.8	0.001*	2.8 ± 3.0	169.1 ± 311.5	0.001*
**TAC score^#^**	618.2 ± 1111.1	306.0 ± 482.4	753.5 ± 1276.3	0.028*	496.2 ± 132.6	749.2 ± 1297.3	0.007*

Values present as mean ± SD or n (%). BAT = Brown adipose tissue; BAT-RS = Brown adipose tissue radiomics score; CAC = Coronary artery calcium; TAC = Thoracic aorta calcium ^#^Only calcium positive was presented, but all patients were accepted for p value calculation. *p value < 0.05. ^a^43 patients from development cohort, 20 patients from external validation cohort 1 ^b^43 control patients from development cohort, 40 control patients from external validation cohort 1 and 2 (n = 20 for each cohort), respectively.

## Abbreviations

AUROC: area under the receiver operating characteristic curve
BAT: brown adipose tissue
CAC: coronary artery calcium
CI: confidence interval
FDG: fluorodeoxyglucose
GLSZM: gray-level size zone matrix
GLCM: gray-level co-occurrence matrix
GLRLM: gray-level run-length matrix
GLDM: gray-level dependence matrix
HU: Hounsfield unit
ICC: inter-class correlation coefficient
LASSO: least absolute shrinkage and selection operator
MRI: magnetic resonance imaging
NGTDM: neighboring gray tone difference matrix
NPV: negative predictive value
OR: odds ratio
PET-CT: positron emission tomography-computed tomography
PPV: positive predictive value
PSM: propensity score matching
ROC: receiver operating characteristic
ROI: region of interest
RS: radiomics score
SUV_bm_: standard uptake value at the basal metabolic rate
SVM: support vector machine
TAC: thoracic aorta calcium
