# Supplementary material for: Development and validation of a nonenhanced CT based radiomics model to detect brown adipose tissue
Source: Theranostics. 2023 Mar 5;13(5):1584–93. doi: 10.7150/thno.81789 (PMC10086200; doi:10.7150/thno.81789)
Supplement: Supplementary file 1 — Supplementary figures and tables. [file thnov13p1584s1.pdf]

Supplementary Materials

Table S1. The details for image filters and their explanation

**Image filter (10 types)**	**Explanation**
**Original**	No filter applied
**Wavelet**	Wavelet filtering, yields 8 decompositions per level (all possible combinations of applying either a High or a Low pass filter in each of the three dimensions.
**LoG**	Laplacian of Gaussian filter, edge enhancement filter. Emphasizes areas of gray level change, where sigma defines how coarse the emphasised texture should be. A low sigma emphasis on fine textures (change over a short distance), where a high sigma value emphasises coarse textures (gray level change over a large distance).
**Square**	Takes the square of the image intensities and linearly scales them back to the original range. Negative values in the original image will be made negative again after application of filter.
**SquareRoot**	Takes the square root of the absolute image intensities and scales them back to original range. Negative values in the original image will be made negative again after application of filter.
**Logarithm**	Takes the logarithm of the absolute intensity + 1. Values are scaled to original range and negative original values are made negative again after application of filter.
**Exponential**	Takes the the exponential, where filtered intensity is e^(absolute intensity). Values are scaled to original range and negative original values are made negative again after application of filter.
**Gradient**	Returns the gradient magnitude.
**LBP2D**	Calculates and returns a local binary pattern applied in 2D.
**LBP3D**	Calculates and returns local binary pattern maps applied in 3D using spherical harmonics. Last returned image is the corresponding kurtosis map.

Table S2. Performance of the radiomics model with different feature selections or different machine learning algorithms in the internal validation cohort.

Feature selections[a]	Sensitivity, %	Specificity, %	PPV, %	NPV, %	Accuracy, %	AUROC (95%CI)
LASSO	76.8	74.7	84.8	63.6	76.1	0.81 (0.75-0.87)
ICC+ LASSO	61.3	81.2	63.9	79.4	74.2	0.78 (0.72-0.85)
Pearson* + LASSO	79.7	72.0	84.0	65.9	77.0	0.81 (0.74-0.87)
ICC + Pearson* + LASSO	89.3	61.6	55.8	91.4	77.9	0.85 (0.79-0.90)
ICC + Pearson* + F Test	68.0	71.0	56.0	80.3	70.0	0.78 (0.72-0.85)
ICC + Pearson* + Tree Based	72.0	67.4	54.6	81.6	69.0	0.75 (0.69-0.82)
**Machine learning algorithms**						
Support Vector Machine	89.3	61.6	55.8	91.4	77.9	0.85 (0.79-0.90)
Logistic Regression	68.0	63.8	50.5	78.6	67.1	0.73 (0.66-0.80)
Gradient Boosting	42.7	84.8	60.4	73.1	70.4	0.74 (0.67-0.81)
AdaBoost	53.3	81.9	61.5	76.4	70.0	0.76 (0.69-0.82)
Linear SVC	54.7	76.8	56.2	75.7	69.0	0.71 (0.64-0.78)
XG Boost	51.7	84.8	64.4	76.0	70.9	0.79 (0.72-0.85)
K-Nearest Neighbors	53.3	81.9	61.5	76.4	71.8	0.74 (0.67-0.81)

Random Forest	36.0	86.2	58.7	71.3	70.4	0.72 (0.65-0.79)
Linear Discriminant Analysis	41.3	86.2	62.0	73.0	70.4	0.73 (0.66-0.80)
Decision Tree	48.0	65.9	43.4	70.0	66.2	0.59 (0.52-0.66)

[a]Models were developed by Support Vector Machine with different feature selections; *Pearson was presented as Pearson's correlation coefficient;

LASSO = least absolute shrinkage and selection operator; ICC = inter-class correlation coefficient; PPV = positive predictive value; NPV = negative predictive value; AUC = area under the curve; CI = confidence interval; SVM = Support Vector Machine

Table S3. Radiomic features selected for model development.

Image filter	Feature group	Feature name
original	shape	Flatness
original	shape	LeastAxisLength
original	shape	Maximum2DDiameterSlice
log-sigma-2-0-mm 3D	firstorder	90Percentile
log-sigma-2-0-mm-3D	firstorder	Kurtosis
log-sigma-3-0-mm-3D	firstorder	Kurtosis
log-sigma-4-0-mm-3D	firstorder	Kurtosis
log-sigma-5-0-mm-3D	firstorder	Range
log-sigma-5-0-mm-3D	firstorder	RobustMeanAbsoluteDeviation
log-sigma-5-0-mm-3D	firstorder	Uniformity
log-sigma-5-0-mm-3D	GLRLM	GrayLevelVariance
log-sigma-5-0-mm-3D	GLRLM	HighGrayLevelRunEmphasis
Logarithm	firstorder	10Percentile
Logarithm	firstorder	Minimum
Logarithm	GLCM	MaximumProbability
Square	GLSZM	ZoneEntropy

lbp-2D	GLSZM	ZoneEntropy
lbp-3D-k	GLRLM	RunLengthNonUniformity
Gradient	GLCM	Inverse Difference
Gradient	GLDM	DependenceNonUniformityNormalized
exponential	GLSZM	SizeZoneNonUniformityNormalized

GLSZM = gray-level size zone matrix; GLCM = gray-level co-occurrence matrix; GLRLM = gray-level run-length matrix; GLDM = gray-level dependence matrix;

Table S4. Comparison of cardiovascular calcification between the BAT-RS and non-BAT-RS group in each cohort

	Model Development Cohort			External-Validation Cohort 1			External-Validation Cohort 2		
**Variables**	**BAT-RS**	**Non-BAT-RS**	**p value**	**BAT-RS**	**Non-BAT-RS**	**p value**	**BAT-RS**	**Non-BAT-RS**	**p value**
**Patients, n**	49	37	-	21	19	-	18	22	-
**CAC > 0, n (%)**	2 (4.1)	7 (18.9)	0.062	0 (0.0)	2 (10.5)	0.219	0 (0.0)	6 (27.3)	0.024*
**TAC > 0, n (%)**	11 (22.4)	12 (32.4)	0.300	2 (9.5)	7 (36.8)	0.092	3 (16.7)	12 (54.5)	0.014*
**CAC volume#, mm^3**	4.0 ± 2.8	296.7 ± 355.7	0.020*	0	24.4 ± 25.5	0.573	0	14.1 ± 13.9	0.018*
**TAC volume#, mm^3**	366.2 ± 491.4	991.2 ± 1171.5	0.220	58.8 ± 67.0	699.8 ± 1565.9	0.117	64.1 ± 48.3	162.2 ± 174.7	0.008*
**CAC score#**	2.8 ± 3.0	339.9 ± 402.6	0.020*	0	28.3 ± 29.7	0.573	0	16.8 ± 19.0	0.018*
**TAC score#**	422.0 ± 538.9	1193.1 ± 1422.5	0.212	67.9 ± 78.2	792.4 ± 1773.7	0.117	71.0 ± 55.6	187.4 ± 201.0	0.008*

Values presented as mean ± SD or n (%). BAT = Brown adipose tissue; CAC = Coronary artery calcium; TAC = Thoracic aorta calcium;

#Only calcium positive was presented, but all patients were accepted for p value calculation.

* p value < 0.05.

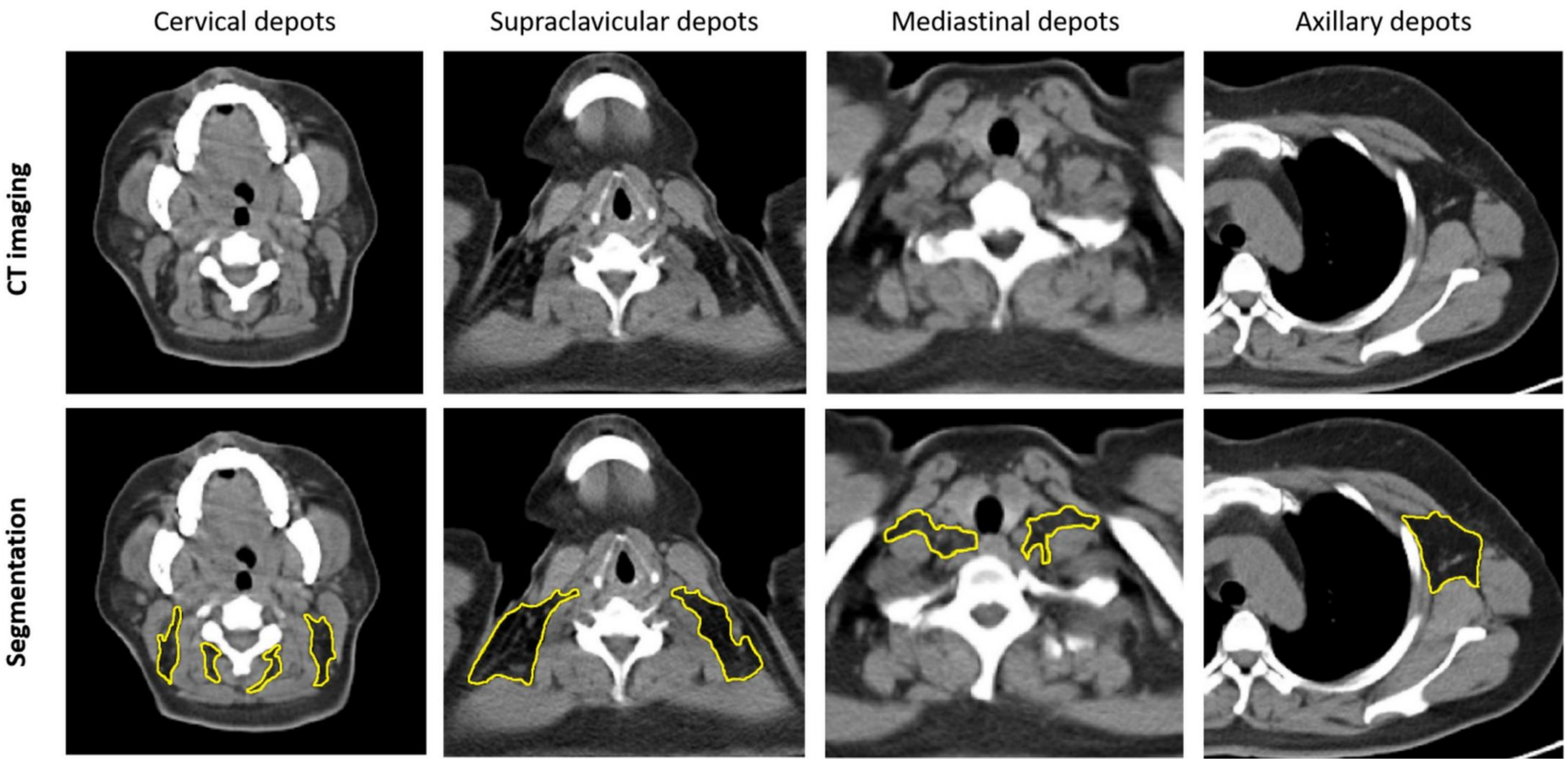

Figure S1. CT image segmentation in cervical, supraclavicular, axillary and mediastinal depots. The depot was defined by anatomic landmarks: cervical (C3–C7), supraclavicular (C7–T3, beside the spine), mediastinal (anterior to the spine, C7–T3), axillary (an extension of the supraclavicular region closed to the thorax, T3–T8. Symmetrical distribution of the depots was determined visually.

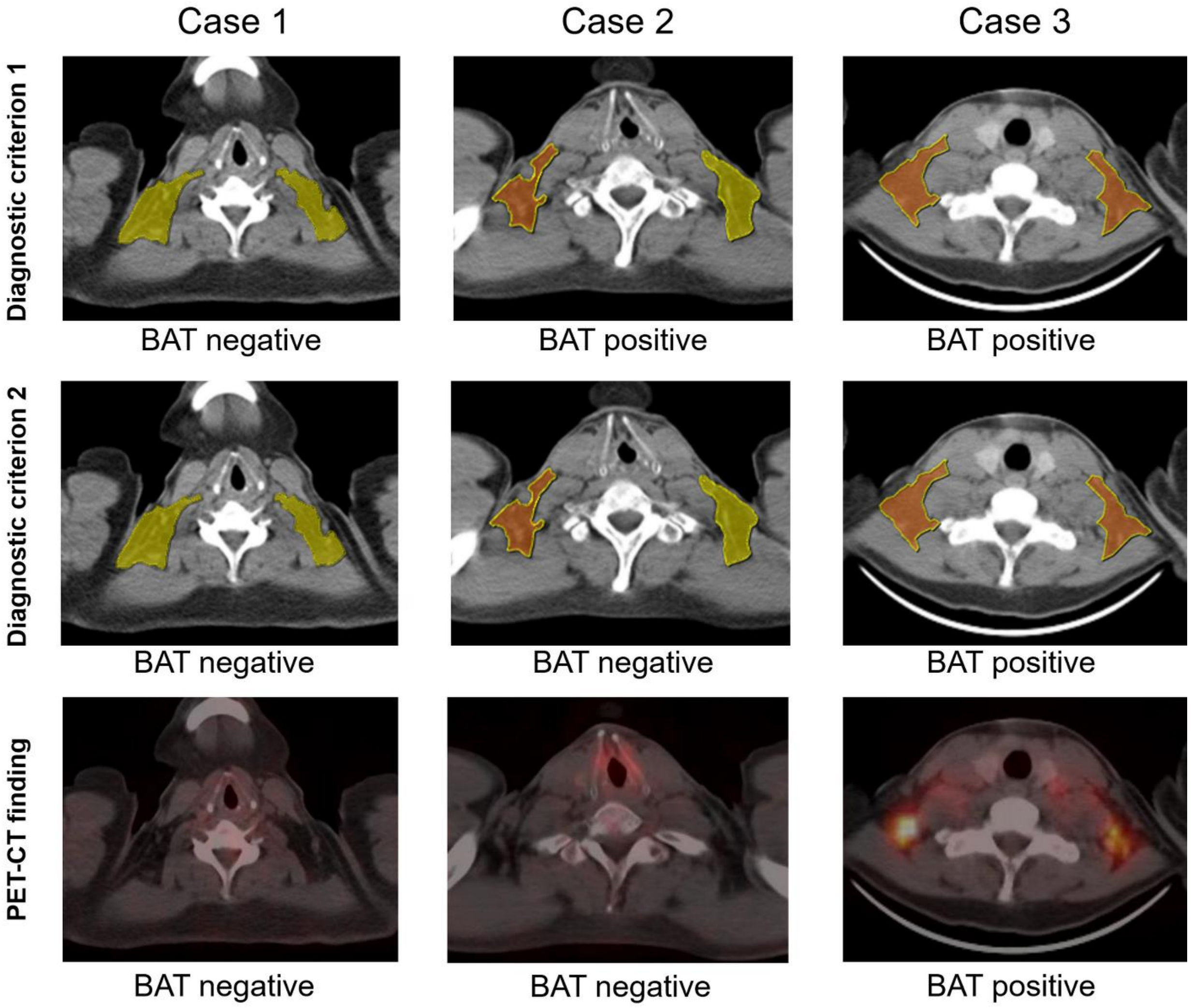

Figure S2. Examples of BAT diagnosis based on the radiomics model on a per patient level using different criteria.

The depot in yellow indicates non-BAT diagnosed by the radiomics model, while the depot in brown indicates BAT diagnosed by the radiomics model. **Case 1** was diagnosed with negative BAT using both criterion 1 and 2 as bilateral depots were non-BAT. **Case 2** was diagnosed as BAT positive in criterion 1 based on the right depot (>0 BAT depot with RS higher than 0.30), but according to criterion 2, it was diagnosed as BAT negative because the BAT depot did not meet the bilateral symmetric distribution standard (Symmetrical distribution of the BAT depots was determined visually). Finally, case 2 had no BAT depot on PET-CT imaging, indicating that the right depot was indeed a false-positive diagnosed by the radiomics portion of the model. **Case 3** was diagnosed as BAT positive using criteria 1 and 2 since bilateral depots were BAT.

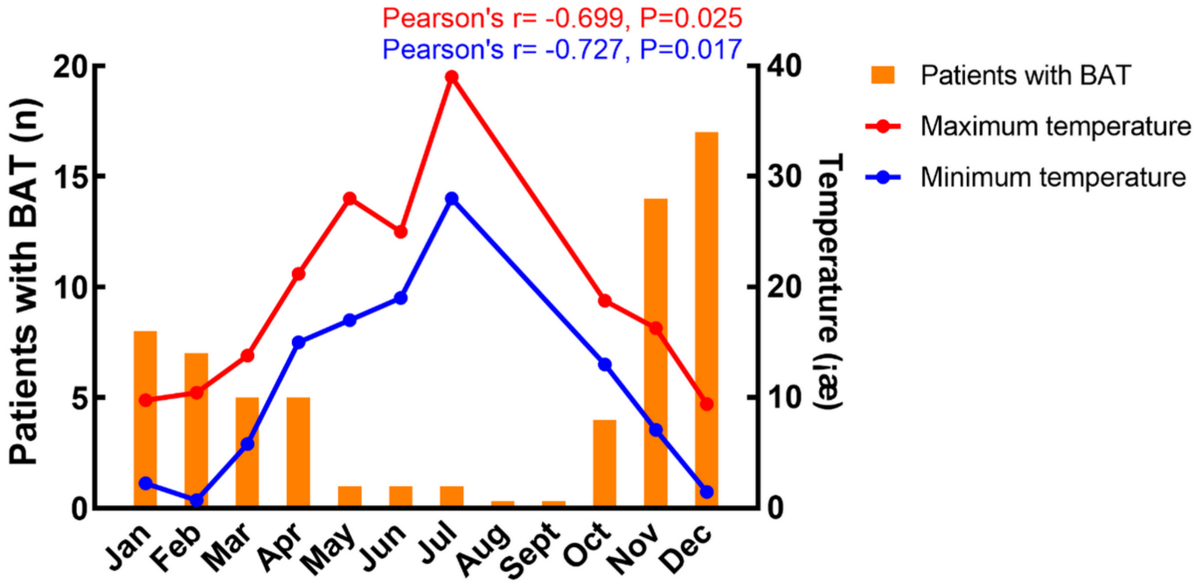

Figure S3. Correlation between the incidence of BAT and the maximum, minimum outdoor temperature at the time of the scan.
